# Human colitis-associated colorectal carcinoma progression is accompanied by dysbiosis with enriched pathobionts

**DOI:** 10.1080/19490976.2025.2479774

**Published:** 2025-03-17

**Authors:** Amr H. Masaadeh, Mohamed Eletrebi, Bishal Parajuli, Nicola De Jager, Dustin E. Bosch

**Affiliations:** a Department of Pathology, Roy J. and Lucille A. Carver College of Medicine, University of Iowa, Iowa City, IA, USA; b Holden Comprehensive Cancer Center, Iowa City, IA, USA

**Keywords:** Colorectal carcinoma, inflammatory bowel disease, microbiome

## Abstract

Dysbiosis and pathobionts contribute to inflammation and the risk of colitis-associated carcinoma (CAC) in animal models, but their roles in humans with this uncommon disease are unknown. We identified microbiome differences in human CAC compared with longstanding inflammatory bowel disease (IBD) and sporadic colorectal carcinoma (CRC). Twenty-four CAC resections were matched with CRC and IBD controls. Methods included histopathology, 16S rDNA metagenomics, and pathobiont-specific qPCR. Beta diversity differed by diagnosis (PERMANOVA *p* = 0.007). The distinguishing taxa included *Akkermansia* enriched in CRC, and *Bacteroides spp*. enriched in IBD. The non-neoplastic mucosae presented distinct beta diversity (*p* = 0.005), but the CAC/CRC tumor microbiomes were similar (*p* = 0.7). Within metastases and margins, Enterobacteriaceae were enriched in CAC, and Bacteroidales in CRC. Pathobiont-specific qPCR confirmed a greater frequency of *pks+ E. coli* and enterotoxigenic *Bacteroides fragilis* in CAC than IBD. High alpha diversity was associated with active inflammation, advanced cancer stage, and shorter overall survival (log-rank *p* = 0.008). Mucosal microbiomes distinguish CAC from longstanding IBD, implicating pathobionts as markers for disease progression. Integrating our findings with prior animal model research, pathobionts promote carcinogenesis in IBD patients through genotoxicity and host cell signaling.

## Introduction

Compared with the general population, inflammatory bowel disease (IBD) patients have greater risk and younger onset age for colorectal cancer.^1^ The incidence of IBD colitis-associated carcinoma (CAC) is related to the duration of IBD and the cumulative degree of inflammation, increasing from ~2% to ~18% at 10 and 30 years, according to a 2001 meta-analysis.^2,3^ More recent data indicate IBD carrier a colon cancer risk 2–3 times greater than the general population.^4^ A greater severity of IBD activity significantly increases the risk for CAC, emphasizing the link between inflammation and carcinogenesis.^5^ Because of the increased risk for colon cancer, surveillance colonoscopy with chromoendoscopy and/or biopsies at every 10 cm interval in the colon is recommended in patients with long-standing (8–10 years) IBD.^6^ Effective inflammation-targeted treatments and surveillance for precursor noninvasive neoplasms (dysplasia) have since reduced the incidence of CAC.^7^ However, the overall survival of patients under 65 years of age with CAC is shorter than that of patients with sporadic colorectal carcinoma (CRC).^8^ The pathology of CAC differs from CRC, with multiple lesions, mucinous features, and high grade being more common.^9^ Known risk factors for CAC include the anatomic extent of disease, stricture, dysplasia in prior biopsies, comorbid primary sclerosing cholangitis, inflammatory polyps, and a family history of CRC.^10^ Longstanding intestinal inflammation in IBD promotes carcinogenesis through complex mechanisms, including STAT3/NF-kB signaling in enterocytes, genomic instability, suppression of anti-neoplastic T cells, and perturbed immune cell interactions with the dysbiotic intestinal microbiome.^11^ CAC progression is modeled as an inflammation, dysplasia, and carcinoma sequence, which has genetic event sequences distinct from those of sporadic CRC.^7,12–14^

CRC is associated with intestinal dysbiosis, including an increased abundance of pathobionts with direct roles in carcinogenesis.^11,15^ Intestinal bacteria and pathobionts are found within primary CRC tumors and distant metastases, where they alter the tumor immune microenvironment.^16^ The enterotoxin fragilysin (genes *bft1-*3) produced by some *B. fragilis* strains (ETBF) cleaves E-cadherin on intestinal epithelial cells and promotes inflammatory cytokine production.^17^ ETBF also causes acute diarrhea, and its detection in fecal specimens has been linked to active inflammation in IBD.^17,18^ The pathobiont *F. nucleatum* increases the risk of metastasis in CRC and may directly contribute to lymphatic or hematogenous spread.^15,19^ High abundances of *F. nucleatum* in metastatic CRC drive resistance to immunotherapy, with impaired CD8^+^ T-cell trafficking in response to bacterium-derived succinic acid.^15,20^ Bacteria in CRC metastases also modulate tumoricidal natural killer cell activity.^19,21^ The polyketide synthase locus (*pks*) in *E. coli* encodes enzymes that synthesize colibactin, a DNA-alkylating genotoxin that produces signature mutations in cancer cells.^22^ Pathobionts appear to have combinatorial effects on carcinogenesis. Adenomas in familial adenomatous polyposis syndrome are frequently associated with *pks+ E. coli* and ETBF, as measured with *in situ* hybridization and PCR.^23^ In support of a causative and potentially synergistic role for these bacteria in carcinogenesis, the cocolonization of mice with both *pks+ E. coli* and ETBF increased tumor multiplicity and shortened survival in a chemical carcinogenesis model (azoxymethane, AOM) compared with noncolonized or monocolonized controls.^23^ Approaches targeting pathobionts and CRC-associated dysbiosis, such as small-molecule inhibition of colibactin synthesis enzymes,^24^ hold therapeutic promise. In summary, several specific bacteria have been causally and mechanistically linked to colorectal carcinogenesis.

Intestinal barrier compromises in IBD may favor pathobiont associations with the mucosa and chronic inflammation.^25,26^ Research with animal models of CAC has highlighted the importance of intestinal bacteria in pathogenesis and the potential for microbiome-targeted therapeutics. Compared with their conventionalized counterparts, germ-free *Il10*^*-/-*^ mice treated with the chemical carcinogen AOM do not develop colon tumors, indicating key roles of the microbiome in CAC development.^27^ The transfer of dysbiotic bacterial communities from *Nod2*-deficient mice was sufficient to confer CAC risk in wild-type mice.^28^ Manipulation of the microbiome can also reduce CAC progression in animal models. Treatment of dextran sodium sulfate (DSS) colitis and AOM mice with oral probiotic strains of *Lactobacillus spp*. reduced tumorigenesis.^29–31^ Probiotic administration of *Bacteroides thetaiotaomicron* in DSS/AOM and xenograft models reduced tumor size.^32^ In summary, commensal bacteria are integral to CAC carcinogenesis, and probiotic administration can ameliorate progression in animal models.

Known CRC-associated pathobionts also contribute to CAC in animal models. In the DSS/AOM model, *F. nucleatum* stimulates β-catenin pathway signaling and epithelial‒mesenchymal transition, in part through interactions between the virulence factor FadA and host E-cadherin.^33,34^ In an *Il10*^*-/-*^ mouse model of intestinal inflammation, the *pks+ E. coli* strain NC101 promoted the development of invasive carcinoma after exposure to AOM.^35^ Deletion of the *pks* locus decreased the number of tumors, depth of invasion, and markers of genotoxin-mediated DNA damage.^35^ The *E. coli pks* island also promotes carcinogenesis in DSS-treated mice deficient in intestinal epithelial cell autophagy (*Atg16l1*^*DIEC*^).^36^ A study of mucosal bacteria in 7 CAC patients highlighted differences from those in 10 CRC patients, with relative enrichment of Pseudomonadota, including the Enterobacteriaceae family.^37^ Although pathobionts such as *pks+ E. coli*, ETBF, and *Fusobacterium nucleatum* have been highly investigated, other intestinal bacteria are likely to contribute to CAC carcinogenesis. A DNA damage screen using bacterial isolates from IBD patients revealed several genotoxins, such as indoleamine from *Morganella morganii*, that increase gut permeability and tumor burden in DSS/AOM-treated gnotobiotic mice.^38^

In summary, causative roles for pathobionts have been established in CAC animal models. Knowledge gaps surround the roles of pathobionts and other commensals in *human* CAC development and progression, in part owing to very limited human specimen-based studies of this uncommon disease. One prior human mucosal microbiome study of CACs (*n* = 7 subjects) concluded that tumor and adjacent microbiomes differ from those of CRC and that the *Fusobacterium* genus has a lower abundance in CAC.^37^ Whether these differences may be related to the chronic inflammation of IBD, treatment differences, or clinicopathologic differences between the CAC and CRC cohorts is not certain.

We designed a retrospective formalin-fixed tissue microbiome profiling study featuring 24 resections of very uncommon human CAC and matched CRC and long-standing nonneoplastic IBD controls. A major goal of this study was to contrast the human CAC microbiome in primary tumors, metastases, and adjacent nonneoplastic mucosa with that in sporadic CRC, emphasizing the detection rates and abundances of known pathobionts. Pathobionts are emphasized because they have well-established mechanisms in carcinogenesis and disease progression in model systems.^33–36,38^ We also investigated microbiome differences between IBD patients who developed CAC and those who did not despite having a similar disease duration. Distinguishing features may identify bacteria contributing to carcinogenesis and be useful for surveillance. Finally, we correlated microbiome features with outcomes of advanced stage and survival in both CAC and CRC.

## Materials and methods

### Study design and case selection

The study protocol was reviewed and approved by the University of Iowa Institutional Review Board (protocol 202,109,011), with a waiver of informed consent. Research methods for human subjects were performed in accordance with the Declaration of Helsinki. CAC resection cases were retrospectively identified using a pathology database and electronic health records. CRC resection controls were selected and matched for tumor location and pathologic stage. Nonneoplastic IBD controls were matched for diagnosis (Crohn’s disease, ulcerative colitis, or indeterminate colitis), disease activity at the time of resection, and time since the initial diagnosis. These matched factors were selected on the basis of prior studies showing their potential influence on the microbiome in IBD. Table 1 contains basic demographics, IBD characteristics, pathology, and treatment information.

**Table 1.** Clinicopathologic features of the CAC, IBD, and CRC cohorts.

	CAC	CRC	IBD	test	p value
**Subjects***n*	24	24	24		
**Demographics**					
gender *% female*	37.5	41.7	33.3	$\chi^{2}$	0.8
age *mean yr, SD*	49 (17)	69 (11)	46 (16)	aov	<0.001
**IBD diagnosis**					
ulcerative colitis *n*	11	*NA*	11	$\chi^{2}$	1
Crohn’s disease *n*	11	*NA*	11		
indeterminate colitis *n*	2	*NA*	2		
**IBD characteristics**					
stricture *n*	3	*NA*	6	$\chi^{2}$	0.3
fistula *n*	2	*NA*	3	$\chi^{2}$	0.6
dysplasia *n*	13	*NA*	2	$\chi^{2}$	<0.001
prior resection *n*	2	0	0	$\chi^{2}$	0.8
disease duration *mean yr, SD*	20 (14)	*NA*	15 (11)	aov	0.1
**IBD activity at resection**				$\chi^{2}$	0.2
severe *n*	8	*NA*	16		
moderate *n*	2	*NA*	2		
mild *n*	5	*NA*	2		
none *n*	6	*NA*	3		
not graded *n*	3	*NA*	1		
**Tumor location**				$\chi^{2}$	0.9
right colon *n*	13	14	*NA*		
transverse/left colon *n*	7	6	*NA*		
rectum *n*	4	4	*NA*		
**Pathologic stage group**				$\chi^{2}$	1
1 *n*	8	7	*NA*		
2 *n*	5	6	*NA*		
3 *n*	4	4	*NA*		
4 *n*	7	7	*NA*		
**Neoadjuvant therapy**					
chemotherapy *n*	6	6	*NA*	$\chi^{2}$	1
radiation therapy *n*	3	3	*NA*	$\chi^{2}$	1
**Adjuvant therapy**					
chemotherapy *n*	8	12	*NA*	$\chi^{2}$	0.2
radiation therapy *n*	0	0	*NA*	*NA*	
**Pathologic response score**				$\chi^{2}$	0.8
0 *n*	1	0	*NA*		
1 *n*	0	1	*NA*		
2 *n*	2	3	*NA*		
3 *n*	3	3	*NA*		
**Medication within last month**				$\chi^{2}$	<0.001
antibiotics *n*	2	2	4		
corticosteroids *n*	3	3	10		
aminosalicylates *n*	7	0	2		
α-TNF *n*	3	0	6		
ursodiol *n*	1	0	0		
azathioprine *n*	1	0	1		
methotrexate *n*	0	0	1		
ustekinumab *n*	0	0	1		
vedolizumab *n*	0	0	3		

Cases were selected and matched based on sex, tumor location, IBD activity, and IBD disease duration. Statistical tests were either Chi-squared ($\chi^{2}$) for categorical data or analysis of variance (aov) for continuous data. Abbreviations: SD standard deviation; yr year; IBD inflammatory bowel disease; CAC colitis-associated carcinoma; CRC colorectal carcinoma.

### Pathology review and scoring

H&E slides for all resections were reviewed for diagnosis, pathologic stage, treatment response score (modified Ryan use for clinical specimens),^39^ and IBD activity score (Nancy Index)^40^ by a board-certified gastrointestinal pathologist (DEB). Tumor-infiltrating lymphocytes were scored according to published criteria^41^ and tumor-infiltrating neutrophils were quantified per high-power field via hot spot analysis.

### 16S rDNA PCR and sequencing

Nucleic acids were extracted from formalin-fixed and paraffin-embedded tissues using the QAIamp DNA FFPE Advanced Kit (Qiagen) with uracil-N-glycosylase to mitigate cytosine deamination. Nonneoplastic tissue from the proximal margins of the rectal resections was selected, and the mucosal layer was dissected. Primary tumors at the bowel luminal surface and metastatic carcinoma in the liver or lungs were also dissected from cancer cases (CAC, CRC). 16S rDNA amplification, library generation, and sequencing were performed as previously described.^42^ Briefly, the V3-V4 region of the 16S rRNA gene was amplified using primers 5’- TCGTCGGCAGCGTCAGATGTGTATAAGAGACAGCCTACGGGNGGCWGCAG-3’ and 5’-GTCTCGTGGGCTCGGAGATGTGTATAAGAGACAGGACTACHVGGGTATCTAATCC-3’. Indexing and library construction were performed using a second round of PCR. Multiplexed samples were sequenced by paired sequencing on a MiSeq instrument (Illumina).

### Microbiome data analysis

16S metagenomic data from nonneoplastic mucosa, tumors, and metastases were separated for processing. The raw sequence reads were processed using QIIME 2.^43^ The sequences were demultiplexed, and the quality profiles were visualized using the demux and summarize functions. The DADA2 pipeline^44^ was used for sequence quality control and feature table generation. Phylogenetic trees were generated using the QIIME 2 phylogeny function. Alpha and beta diversity metrics with group significance statistics were calculated using the q2-diversity plugin in QIIME 2. Taxonomic units were classified using a classifier trained on the Greengenes database (gg_12_8).^45^ Differential abundance testing by linear discriminant analysis (LDA) was performed with LEfSe using a cutoff LDA score of >2.0.^46^ The analysis outputs were visualized via QIIME 2 View (view.qiime2.org) and GraphPad Prism 9 (La Jolla, CA, USA). Metabolic pathways were predicted on the basis of taxonomy and relative abundance using PICRUSt2^47^ and relative enrichment was detected using LDA.

### Low-biomass controls and decontamination

In contrast with fecal metagenomics, microbiome profiling from formalin-fixed paraffin-embedded (FFPE) tissue presents unique challenges. Despite this, prior research has demonstrated that bacterial communities can be accurately profiled from this matrix.^48–51^ Among the challenges are the predominance of human DNA extracted from tissue, DNA damage and cross-linking related to formalin fixation, and the potential for contaminating bacterial DNA during nonsterile specimen handling. Importantly, these features are true of *all* FFPE biospecimens which are handled uniformly in pathology; therefore systematic biases among the study groups of this work are not expected. Controls included 16S rDNA amplification and sequencing from tissue-free FFPE blocks and reagents only. We applied several additional “decontamination” approaches: 1) exclusion of reads mapping to taxonomic groups that are known contaminants from FFPE processing at our institution, identified by a previous 10-subject intraindividual FFPE and fresh frozen tissue comparison; 2) statistical identification of likely contaminants with the *decontam* package in R^52^; and 3) exclusion of taxonomic groups that are not identified in at least 1% of high-quality fecal metagenomes from the Human Microbiome Project^53^ as measured with MetaPhlAn.^54^ After decontamination, there was a median of 14,361 high-quality reads per sample (interquartile range 7,048–23,975), which is comparable in sequencing depth to previous studies of colon FFPE samples.^49^

### Pathobiont gene quantitative PCR

Multiple qPCR primer pairs were designed for targeting *F. nucleatum nusG* and 16S rDNA, the *B. fragilis* enterotoxin gene *bft1*, and the *E. coli pks* locus genes *clbB* and *cnf1*. Total bacteria were quantified with primers targeting the V7 region of 16S rDNA and human DNA using the actin gene. The primers were bioinformatically filtered for off-target effects using BLAST, NCBI databases, and a large collection of metagenome-assembled genomes (MAGs).^55^ qPCR was performed using SYBR Green and thermal melt analysis to confirm the expected amplicon. Control reactions were performed on DNA extracted from pure pathobiont cultures. Pathobiont detection in biospecimens was defined as a C_T_ value of less than 35 cycles and a thermal melt curve similar to that of pure culture controls. Relative quantities were calculated via the 2^−∆Ct^ method^56^ with total bacteria (16S) used as the reference. All the qPCR primer sequences are listed in Supplementary Table 1.

### Statistical analysis

Several alpha diversity metrics, including Shannon’s index, Simpson’s index, Faith’s PD, and Chao1, were derived with QIIME2. Several metrics have been employed because of their distinct sensitivity to microbiome alterations.^57^ The alpha diversity plots are representative of these multiple analyses, which showed consistent results in the statistical tests for our study. Statistical tests for alpha diversity were nonparametric, either the Kruskal‒Wallis test or the Mann‒Whitney U test, depending on the number of groups compared, and were performed using Prism (GraphPad). For beta diversity analysis, a single metric of Bray‒Curtis distance was chosen before analysis and was calculated using QIIME2. Beta diversity was visualized using Bray-Curtis distance metrics and principal component analysis, which included all the detected operational taxonomic units. Statistical testing for global differences across groups was PERMANOVA performed in QIIME2.^43^ For linear discriminant analysis, a cutoff score of 2.0 was selected before analysis according to the default settings of LefSe.^46^ Recurrence-free survival was calculated as the date of recurrence or death minus the date of resection with censored data. Kaplan‒Meier curves for subjects with high and low alpha diversity (bitiles) were compared using the Mantel‒Cox log-rank test. Quantitative PCR detection rates were compared with Fisher exact tests, and relative gene quantities were compared with nonparametric Kruskal‒Wallis tests.

## Results

### Defining a CAC cohort with matched controls

Several known factors, such as the dysbiosis of IBD, Crohn’s disease (CD) *vs*. ulcerative colitis (UC) diagnosis, long-standing inflammation, the presence of a neoplasm, stage, and chemo/radiation therapy, modulate the intestinal microbiome in CAC patients. A comparison with “healthy controls” would identify microbiome differences but create uncertainty about the relationship with neoplasms, IBD, or therapy. We therefore matched 24 CAC cases and CRC controls for anatomic location and pathologic stage and 24 long-standing nonneoplastic IBD resections. The CAC and CRC comparison groups also had similar adjuvant and neoadjuvant therapies and pathologic responses to neoadjuvant therapy (Table 1). The CRC subjects were significantly older on average. To focus the study on CAC-specific changes not simply due to the underlying IBD, the 24 nonneoplastic IBD controls were matched for diagnosis, disease duration, and inflammation activity. Not surprisingly, precursor intraepithelial neoplasms (dysplasia) were more common in prior biopsies from CAC subjects as they progressed along the dysplasia-carcinoma sequence (Table 1). Four CAC and 6 CRC subjects had resection of distant metastases, and these biospecimens were included in the study. The cohort design takes full advantage of testing archived FFPE tissue to construct a sizable CAC cohort and select matched controls from the more numerous CRC and IBD resections. Microbiome analyses of this cohort were expected to address two primary questions: (1) How does the microbiome differ between long-standing IBD patients who develop CAC and those free of neoplasms? (2) How do CAC microbiomes differ from those of CRC, independent of stage and location?

### Beta diversity differs by diagnosis and site

**Figure 1.**
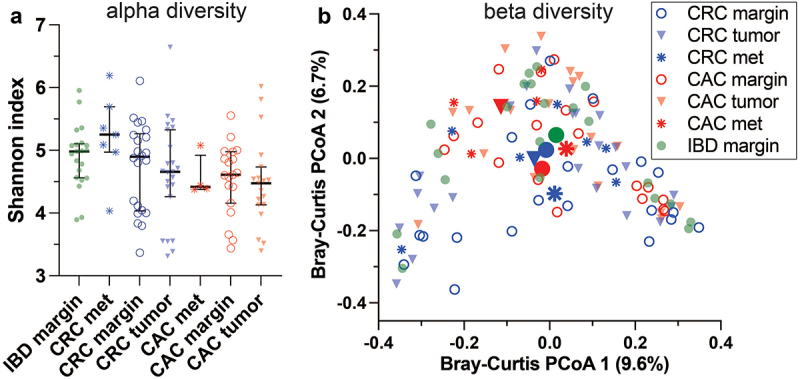
Global microbiome diversity comparison among colitis-associated carcinoma, sporadic colorectal carcinoma, and non-neoplastic inflammatory bowel disease. Bacteria were profiled from FFPE biospecimens using 16S rDNA sequencing. a) Alpha diversity did not differ significantly by site or diagnosis. Kruskal–Wallis test *p* = 0.08. b) Bray-Curtis distance principal component analysis highlighted significant beta diversity differences by site and diagnosis. PERMANOVA test *p* = 0.007. Larger filled shapes indicate calculated centroids. Abbreviations: CAC, colitis-associated carcinoma; CRC, colorectal carcinoma; IBD, inflammatory bowel disease; met, metastasis; PCoA principal component analysis.

**Figure 2.**
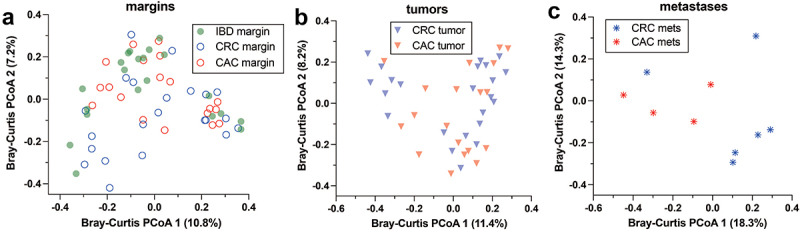
Microbiome differences at nonneoplastic mucosal margins, tumors, and distant metastases. a-c) Bray-Curtis distance principal component analysis revealed significant differences at margins (PERMANOVA test *p* = 0.005) and metastases (PERMANOVA test *p* = 0.046). CRC and CAC tumor microbiomes did not differ significantly (PERMANOVA test *p* = 0.7). Abbreviations: CAC, colitis-associated carcinoma; CRC, colorectal carcinoma; IBD, inflammatory bowel disease; PCoA principal component analysis.

Bacterial 16S rDNA metagenomes were measured for all 72 cases, which were sampled separately at a nonneoplastic mucosal margin, the primary tumor (for CAC and CRC), and any available distant metastases. Several alpha diversity metrics did not differ significantly by diagnosis or site (Figure 1a). Alpha diversity tended to be lower in CAC subjects than in CRC subjects at all sites, but the difference was not statistically significant. Perhaps, the most surprising alpha diversity finding is that it is maintained at distant metastatic sites compared with the luminal gut. The global beta diversity of all the metagenomes differed by study group and site, as measured with Bray‒Curtis distances and principal component analysis (Figure 1b, PERMANOVA *p* = 0.007). The microenvironments of nonneoplastic mucosa (margin), tumors and metastases in distant organs are likely different in several respects, such as available bacterial nutrients, leukocyte distributions, and local oxygen concentrations. We stratified the global beta diversity analysis by site (Figure 2). CAC and CRC primary tumors did not differ significantly, but nonneoplastic mucosae and distant metastases were distinct (PERMANOVA *p* = 0.005, 0.046). The findings highlight the beta diversity features of CAC, which are distinct from those of both CRC and IBD, not dependent on the clinicopathologic characteristics listed in Table 1.

### Enterobacteriaceae are enriched in CAC, and bacteroidales are enriched in CRC

**Figure 3.**
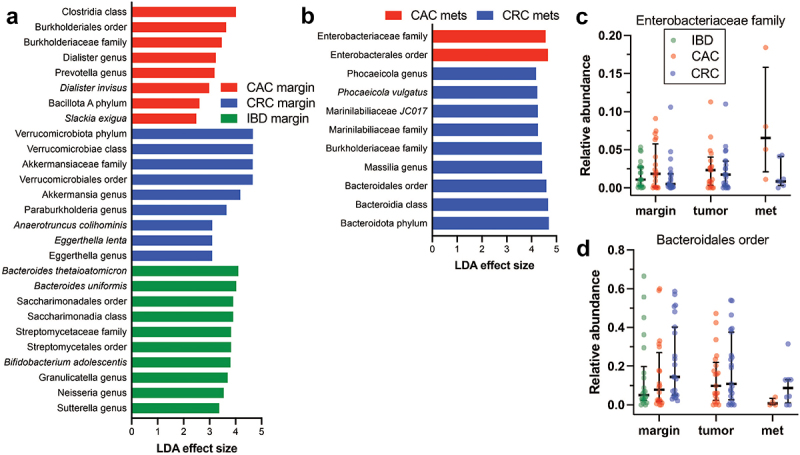
Specific taxon enrichment by diagnosis and site. Linear discriminant analysis of nonneoplastic mucosa (a) identified taxonomic group enrichment by study group. CAC had prominent clostridia and burkholderiaceae, CRC was enriched for Akkermansia and related higher taxonomic groups, and IBD had high *Bacteroides spp.*, among others. (b) In distant metastases, Enterobacteriaceae were enriched in CAC subjects, whereas bacteroidales were enriched in CRC. Abbreviations: CAC, colitis-associated carcinoma; CRC, colorectal carcinoma; IBD, inflammatory bowel disease; met, metastasis.

Since beta diversity differs between nonneoplastic mucosal margins and metastases, we assessed specific taxon differences with LefSe linear discriminant analysis^46^ (Figure 3). The nonneoplastic mucosa of CAC had relative enrichment of Clostridia and Burkholderiaceae. In contrast, CRC margins were characterized by increased abundance of Verrucomicrobiota, including *Akkermansia*. *Bacteroides uniformis* and the probiotics *Bacteroides thetaiotaomicron* and *Bifidobacterium* were most among the most distinct in nonneoplastic IBD subjects. Our findings, together with those of prior animal model studies, are compatible with the possibility that these bacteria are protective against CAC development in IBD patients.

When CAC and CRC distant metastases were compared, Enterobacteriaceae were enriched in CAC, whereas Bacteroidales were more abundant in CRC (Figure 3b). Relative abundance plots of these two taxonomic groups (Figure 3c,d) highlight trends of more Enterobacteriaceae at all sites in CAC, in contrast with more Bacteroidales in CRC. Since these taxonomic groups include *pks+ E. coli* and ETBF, the findings suggest that pathobionts may be differentially abundant in CAC and CRC. Alternatively, different organisms within these taxonomic groups may underly the enrichment patterns. We tested these possibilities with qPCR.

### CAC and CRC microbiomes are enriched in pathobionts, with pks+ E. coli occurring more frequently in CAC metastases

**Figure 4.**
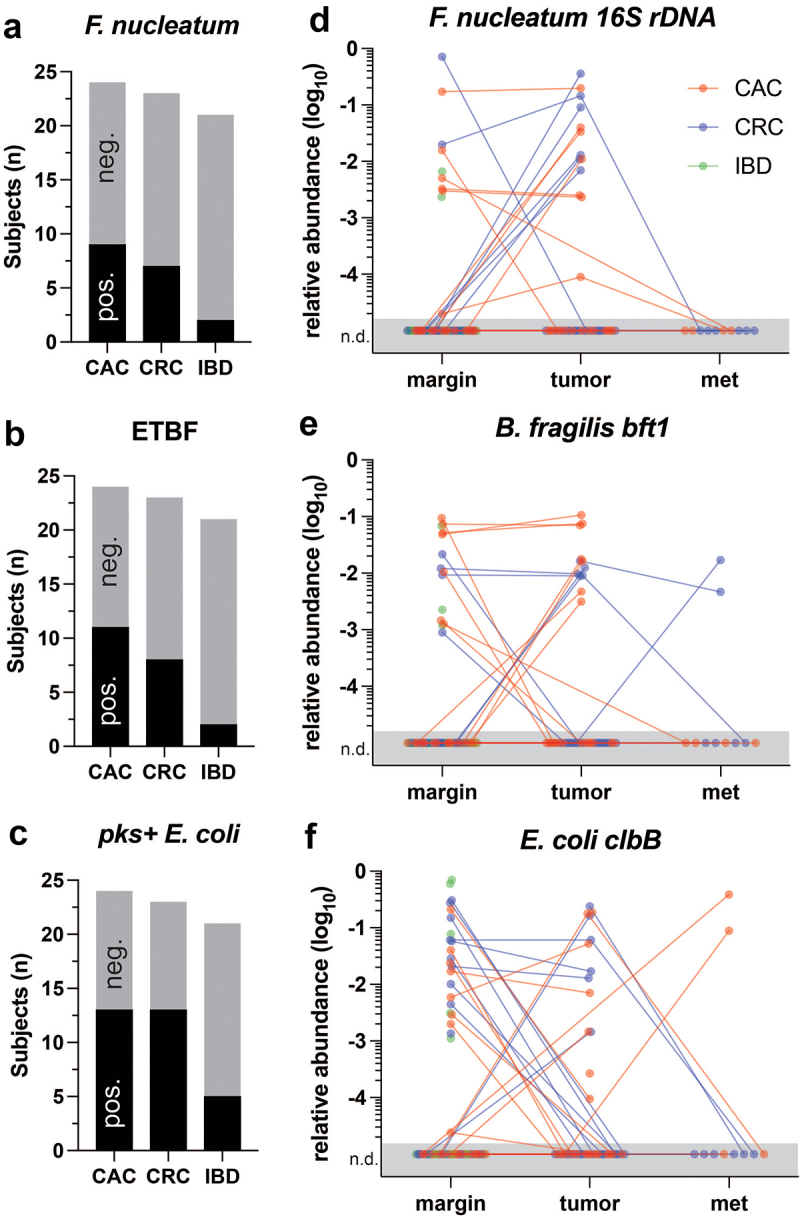
*Pathobiont quantitation in colitis-associated carcinoma, sporadic colorectal carcinoma, and long-standing nonneoplastic IBD*. a-c) Pathobiont-specific qPCR detection counts visualized as bar graphs showed a greater frequency of the pathobionts *F. nucleatum* (p = 0.08), ETBF (p = 0.054), and pks+ *E. coli* (p = 0.03) in both CAC and CRC subjects than in IBD only subjects. *p* values represent fisher’s exact tests. d-f) Pathobiont relative abundances (compared with total bacterial 16S) were plotted with lines connecting biospecimens from the same subject. Pathobiont abundances were dynamic by site and were frequently detectable in tumors but not margins or vice versa. Consistent with the 16S metagenomics results, ETBF was more abundant in CRC and pks+ *E. coli* in CAC metastases. No differences in the qPCR abundances stratified by site were statistically significant between the study groups. Abbreviations: CAC, colitis-associated carcinoma; CRC, colorectal carcinoma; ETBF, enterotoxigenic *bacteroides fragilis*; IBD, inflammatory bowel disease; met, metastasis.

16S rDNA sequencing does not have sufficient strain resolution to distinguish pathobionts, such as *pks+ E. coli* and ETBF, which are defined by one or a few accessory genes. To directly detect and quantify pathobionts, qPCR with primers targeted to *F. nucleatum*, ETBF, and *pks+ E. coli* (Table S1) was applied to all biospecimens with sufficient available DNA (Figure 4). All three pathobionts were more frequently detected in the microbiomes of CAC and CRC subjects than in those of long-standing nonneoplastic IBD subjects. *pks+ E. coli* were detected at similar rates in human CAC and CRC. Pathobiont relative abundance differences did not reach statistical significance at any site. However, ETBF was detected in 2 of the 6 CRC distant metastases (33%), and no CAC metastasis was detected. Conversely, *pks+ E. coli* colonized 2 of the 4 (50%) CAC and no CRC metastases. These findings align with the 16S metagenomics data (Figure 3b) and further suggest differences in specific pathobionts between distant metastatic CRC and CAC.

### Tumor-infiltrating lymphocytes and neutrophils do not differ between CAC and CRC

The chronic and active inflammation associated with inflammatory bowel disease and the different underlying genetic sequences may be expected to produce distinct tumor immune microenvironments (TiME) in CAC and CRC. We therefore investigated tumor-infiltrating lymphocytes (TILs) and neutrophils in CAC *vs*. CRC subjects in our stage-matched cohort and further asked whether these immune cells are related to bacterial taxonomic abundances.

**Figure 5.**
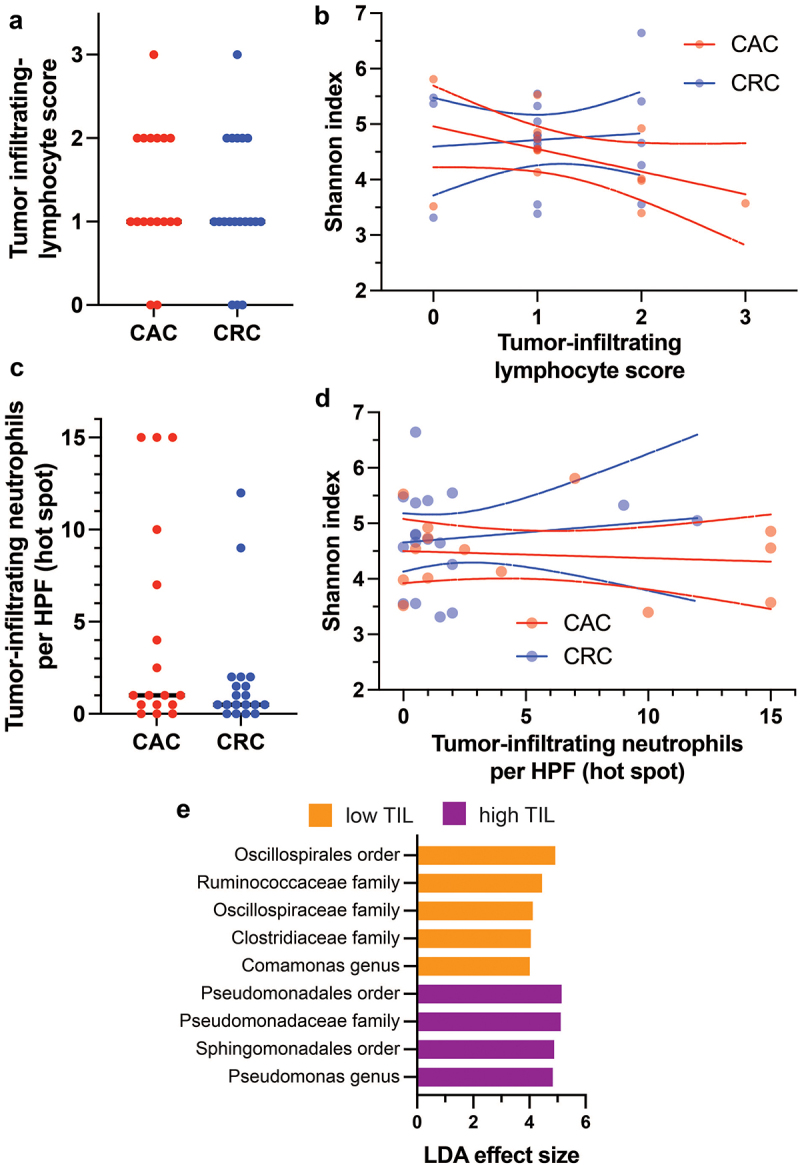
Comparison of primary tumor infiltrating immune cells. Tumor-infiltrating lymphocytes (TILs) (a, b) and neutrophils (c, d) were similar in CAC and CRC subjects (Mann-Whitney tests *p* = 0.6 and *p* = 0.3) and were not significantly related to microbiome alpha diversity. The dotted lines represent 95% confidence intervals for linear regression. (e) Linear discriminant analysis identified taxonomic groups associated with high or low TIL (scores 2–3 *vs*. scores 0–1). No significant taxonomic differences were related to neutrophils. Abbreviations: CAC, colitis-associated carcinoma; CRC, colorectal carcinoma.

Tumor-infiltrating lymphocyte scores and neutrophil counts were not significantly different between CAC and CRC primary tumors (Figure 5). These specific TiME features are similar in CAC and CRC, which is consistent with the lack of significant microbiome differences in primary tumors (Figure 2b). We next asked whether infiltrating lymphocytes or neutrophils were related to microbiome diversity. Alpha diversity (Shannon index) was not significantly associated with either TiME feature or differences between CAC and CRC (Figure 5). Linear discriminant analysis was conducted based on grouping of tumor microbiomes by high or low (bitiles) tumor-infiltrating neutrophils or lymphocytes. No taxa were significant in the neutrophil analysis. High TILs were associated with increased *Pseudomonas spp*. and low TILs with increased Oscillospiraceae and Ruminococcaceae, among other taxa (Figure 5e). The TIL-discriminating taxa exhibited little overlap with those distinguishing CAC, CRC, and IBD (Figure 3). Taken together with the lack of TIL differences in CAC *vs*. CRC, these findings do not provide compelling evidence that the measured TiME/microbiome interactions are different in CAC and CRC.

### Bacterial pathway analysis identifies links to disease pathogenesis and treatment response

**Figure 6.**
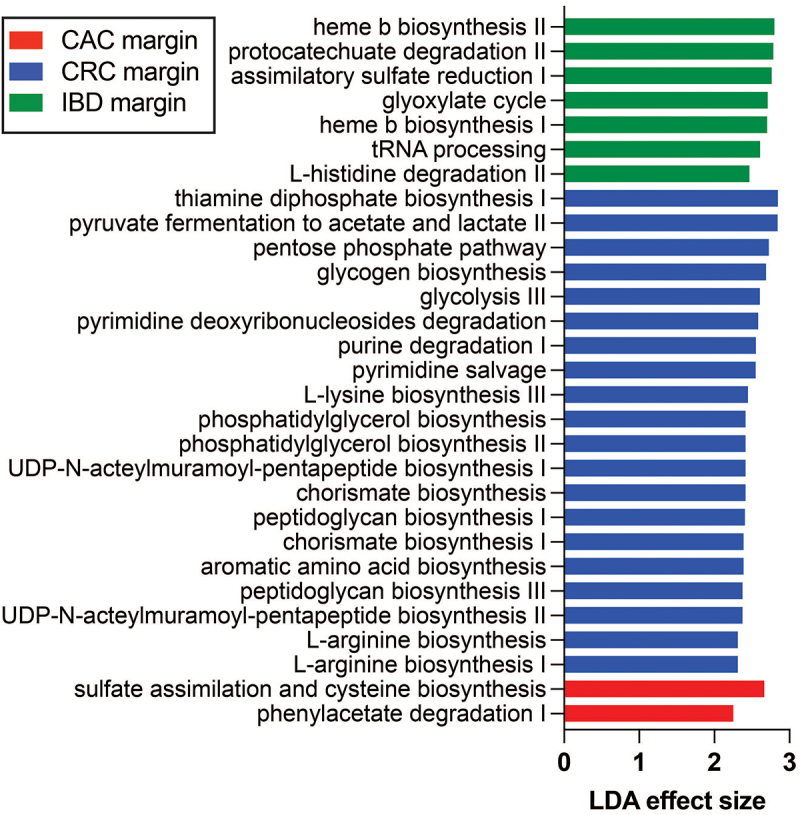
Bacterial gene function pathway enrichment in the nonneoplastic mucosal microbiomes of CAC, CRC, and IBD. Heme biosynthesis was increased in IBD microbiomes, which may be needed for protection from reactive oxygen species related to active inflammation. CRC microbiomes were enriched in several nucleotide biosynthesis pathways, which may reflect selective survival during 5-fu-based chemotherapy. CAC is enriched with bacteria that assimilate sulfate for cysteine biosynthesis. Abbreviations: CAC, colitis-associated carcinoma; CRC, colorectal carcinoma; IBD, inflammatory bowel disease.

Since there was a robust difference in the non-neoplastic mucosal microbiomes of IBD, CRC, and CAC subjects (Figures 1–2), we sought functional capacities enriched in these different microbiomes. Functional metagenome prediction with PICRUSt2^47^ identified several pathways enriched in each study group (Figure 6). The IBD microbiome has a greater capacity for heme biosynthesis, which is known to be important for reactive oxygen species neutralization in the context of neutrophilic inflammation. The CAC microbiome presented increased sulfate assimilation and cysteine biosynthesis capacity, which is also related to H_2_S metabolism. This finding also suggests that bacterial cysteine production may be an important source of glutathione, whose reductive capacity is critical for tumor growth and progression.^58^ The mucosal microbiomes of CRC subjects were enriched for several pathways integral to nucleotide metabolism. One explanation for this could be the effects of 5-FU-based chemotherapy.

### Nonneoplastic mucosal microbiome alpha diversity predicts recurrence-free survival in patients with CAC and CRC

**Figure 7.**
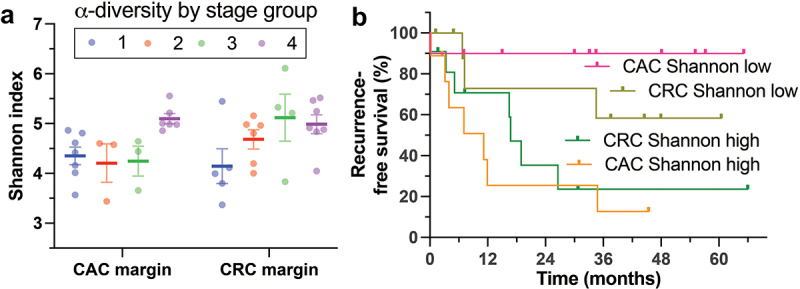
Correlation of alpha diversity in the nonneoplastic mucosa with stage group and survival outcomes. a) Mucosal microbiome alpha diversity was greater in both CAC and CRC subjects with advanced-stage cancer, particularly those with distant metastasis at the time of biospecimen collection (stage group 4). Two-way ANOVA test *p* = 0.02. b) High nonneoplastic mucosal alpha diversity (bitiles) predicted shorter recurrence-free survival in both CRC and CAC subjects. Log-rank (mantel-cox) test *p* = 0.009. Abbreviations: CAC, colitis-associated carcinoma; CRC, colorectal carcinoma.

Microbiome components, including pathobionts, have been implicated in CRC disease progression in animal models.^15,19^ We asked whether intestinal microbiome features are correlated with stage and survival outcomes and whether CAC and CRC differ in this regard. Since CRC and CAC tumors had similar microbiomes and TiME features in the preceding experiments, we focused on the nonneoplastic mucosal (margin) microbiome, which is likely more closely related to the fecal microbiome. High alpha diversity according to several metrics (Shannon diversity shown in Figure 7) was correlated with advanced stage. While the Shannon index was high in both CAC and CRC subjects with distant metastases at the time of resection (stage IV), modest differences in CAC and CRC stage groups II-III alpha diversity were indicated by a 2-way ANOVA interaction factor with a *p* value of 0.02 (Figure 7a).

Alpha diversity is related to stage group, and we reasoned that it may also predict survival outcomes. The CAC and CRC groups were divided into high-alpha diversity and low-alpha diversity groups (bitiles) and compared with the outcome of recurrence-free survival (Figure 7b). Consistent with the stage group correlation, high alpha diversity predicted shorter recurrence-free survival in both CAC and CRC subjects (Mantel‒Cox log-rank *p* = 0.009). We conclude that high mucosal microbiome alpha diversity away from the primary tumor (at margins) is correlated with advanced stage at resection and shorter survival in both CAC and CRC patients.

## Discussion

The central finding of this study is that the human CAC mucosal microbiome differs significantly from that of both stage-matched CRC and long-standing nonneoplastic IBD. Pathobionts, including the seminal genotoxic bacterium in inflammatory colitis mouse models, *pks+ E. coli* ,^35,59^ are enriched in CAC compared with IBD but are not significantly different from those in CRC. Pathobionts have well-established mechanisms in carcinogenesis and disease progression in model systems.^33–36,38^ What this study adds to prior knowledge is an essential link between these mechanisms, previously described in animal and cell culture models, and human CAC using clinical biospecimens. Tumor-infiltrating leukocytes and the tumor microbiome were not distinguishable between CAC and CRC, indicating that these aspects of the tumor microenvironment are similar regardless of cancer etiology. Assimilatory sulfate reduction is enriched in IBD and is a major pathway to H_2_S production by intestinal bacteria, a behavior previously linked to IBD pathogenesis.^60^ Microbiome alpha diversity in the nonneoplastic mucosa is positively correlated with CRC/CAC stage, and a high Shannon index is a predictor of significantly shorter recurrence-free survival.

The retrospective design of this study using FFPE tissue allowed the construction of the largest CAC microbiome cohort to date and controlled critical potential confounders through matching, such as cancer stage, anatomic location, and the length and severity of IBD-related inflammation. Most of the very uncommon CAC cases arise in the context of 8+ years of chronic and active inflammation and are usually treated with several IBD medications, all of which are likely to affect the microbiome. In contrast to typical prospective microbiome studies comparing to “healthy controls”, this study design enables comparison to a similarly treated IBD cohort with a mean disease duration of 15 years (Table 1). Because of this study design, we can conclude that mucosal microbiome differences between CAC and CRC are not simply due to the known dysbiosis of long-standing IBD.

Pathobionts have well-established causal roles in carcinogenesis and disease progression in animal models of CAC, such as DSS-AOM.^27,33,35^ Specifically, *pks+ E. coli* produces colibactin, which enhances carcinogenesis in multiple CAC models^35,61^ by modifying host DNA. The relevance of this genotoxic mechanism to human disease is supported by the detection of signature APC mutations in human CRCs colonized by *E. coli* .^22^ The three pathobionts examined in this study, as well as other tumor microbiome components, are linked to the amount, activity, and spatial distributions of immune cells in CRC.^11,15,16^ Owing to the rarity of CAC, human studies with sufficient power to compare pathobiont frequencies in CAC, CRC, and IBD have been lacking. The results of our study revealed pathobiont enrichment in both carcinoma groups compared with the IBD group, which indicates roles for genotoxin producers and other pathobionts in CAC but not necessarily differences from sporadic colon cancer. In fact, several lines of evidence indicate that CAC and CRC tumors have similar microenvironments: 1) no significant differences in microbiome global diversity, 2) no significant differences in tumor-resident pathobiont detection rates, and 3) no detectable differences in lymphocyte or neutrophil infiltration patterns. In contrast, the distant metastasis microbiomes of CAC subjects differed significantly from those of CRC subjects (Figure 2c). Although the study was not powered for pathobiont detection analyses in distant metastases, the metagenomic LDA analysis (Figure 3b) and pathobiont qPCR data support more abundant *pks+ E. coli* in CAC metastases, in contrast with more ETBF in CRC metastases.

This study also links the mucosal microbiome at the time of colon cancer resection with the long-term outcome of recurrence-free survival. Importantly, this finding is correlational and does not necessarily imply that high microbiome alpha diversity causes high stage and short survival. Advanced-stage CRC/CAC may have more extensive colonic epithelial barrier disruption and is more likely to be treated with neoadjuvant therapy. The known antibacterial effects of 5-FU by DNA damage may select for bacteria with robust DNA repair and nucleotide metabolism pathways.^62,63^ These differences, among others, may well underlie the differences in mucosal alpha diversity. However, this correlational pattern of stage and survival outcomes could be a useful prognostic marker.

Our study highlights several considerations of how the microbiome may be manipulated to reduce CAC risk in the IBD population. Like CRC, the reduction or elimination of pathobionts is hypothesized to reduce cancer risk, and recent progress toward pathobiont inhibitors is encouraging in this regard.^24,64^ Conversely, some of the taxa enriched in long-standing IBD patients who did not develop CAC may be plausible probiotics, such as *Bacteroides thetaiotaomicron* and *Bacteroides uniformis*. Among the LefSe-detected strains associated with nonneoplastic IBD, *B. thetaiotaomicron* and *Bifidobacterium* have been shown to protect against CAC in animal models when used as probiotics.^32,65^ Probiotic *B. thetaiotaomicron* has been incorporated into clinical trial formulations for IBD patients.^66^ Finally, microbiome features distinguishing CAC and nonneoplastic IBD may also be useful prognostic and predictive markers for the surveillance of neoplasms such as IBD.

### Limitations

A limitation of this study is the timing of biospecimen collection at surgical resection. In all CAC and CRC cases, the neoplasm is fully developed, which precludes definitive assessment of whether microbiome changes, such as pathobiont enrichment, *precede* CAC development and are therefore likely to be targets for surveillance testing. Further studies focusing on time course biospecimens before CAC development may address this limitation and are underway.

A second limitation is variation in treatments. Chemotherapeutics, including the cornerstone colorectal cancer 5-FU treatments, are known to modestly influence the gut microbiome, and the likely effects of neoadjuvant chemotherapy on the nonneoplastic mucosal microbiome are supported by increased nucleotide biosynthesis in the CRC group (Figure 6). However, the neoadjuvant treatment rates were the same in the CRC and CAC groups (Table 1), which greatly reduces the risk of confounding bias. Similarly, IBD patients are treated with a variety of immune-modulating medications that may influence the microbiome. Potential confounding effects of these medications are mitigated by substantial overlap with the CAC population (Table 1). It is not feasible to eliminate medications as potential confounders because CAC is rare, and the selection of complex treatment regimens is dependent on individual responses. Untreated IBD progressing to CAC over 8+ years of disease is exceedingly rare, precluding the evaluation of subjects without prior medications.

Additional studies are needed to address other known CRC/CAC pathobionts, and the selection of three pathobionts for this study was not intended to be exhaustive. Similarly, the TIL and neutrophil scoring approaches, which are based on H&E, are well established but relatively crude measures of the TiME. More detailed TiME analyses, such as T-cell subtyping, spatial analysis, and antitumor immunity marker analysis, may reveal differences between CAC and CRC and/or corresponding microbiome effects.

## Supplementary Material

CAC_table_S1_2.docx

## Data Availability

The dataset supporting the conclusions of this article is available in the NCBI Sequence Read Archive (SRA) under BioProject accession number PRJNA1170223.

## Conference presentation

Part of this study was presented at the United States and Canadian Academy of Pathology Annual meeting, Baltimore MD, March 2024.
