# Supplementary material for: Human colitis-associated colorectal carcinoma progression is accompanied by dysbiosis with enriched pathobionts
Source: Gut Microbes. 2025 Mar 17;17(1):2479774. doi: 10.1080/19490976.2025.2479774 (PMC11917176; doi:10.1080/19490976.2025.2479774)
Supplement: CAC_table_S1_2.docx [file KGMI_A_2479774_SM8539.docx]

| **taxon** | **gene** | **primer name** | **sequence** |
| --- | --- | --- | --- |
| *pks+ E. coli* | *cnf1* | cnf1_fw | GGGGGAAGTACAGAAGAATTA |
| *pks+ E. coli* | *cnf1* | cnf1_rv | TTGCCGTCCACTCTCACCAGT |
| *pks+ E. coli* | *clbB* | clbB10_fw | GTCGTGGAAATGCGTCAGAA |
| *pks+ E. coli* | *clbB* | clbB10_rv | CTGACGGCGACAAGTTACAG |
| ETBF | *bft1* | ETBF_1_fw | GAGCCGAAGACGGTGTATGTGATTTGT |
| ETBF | *bft1* | ETBF_1_rv | TGCTCAGCGCCCAGTATATGACCTAGT |
| ETBF | *neu* | ETBF_2_fw | GCCGGTCAGAATGGGAGTAGGAGACC |
| ETBF | *neu* | ETBF_2_rv | CCCGACCCGGACCTTGCAACAGA |
| *F. nucleatum* | *nusG* | Fn_1_fw | CAACCATTACTTTAACTCTACCATGTTCA |
| *F. nucleatum* | *nusG* | Fn_1_rv | GTTGACTTTACAGAAGGAGATTATGTAAAAATC |
| *F. nucleatum* | 16S rDNA | Fn_10_fw | AAGCGCGTCTAGGTGGTTATGT |
| *F. nucleatum* | 16S rDNA | Fn_10_rv | TGTAGTTCCGCTTACCTCTCCAG |
| Eubacteria | 16S rDNA V7 | V7_fw | GTGGTGCACGGCTGTCGTCA |
| Eubacteria | 16S rDNA V7 | V7_rv | ACGTCATCCACACCTTCCTC |
| Eubacteria | 16S rDNA V3-V4 | 16S_fw | TGTATAAGAGACAGCCTACGGGNGGCWGCAG |
| Eubacteria | 16S rDNA V3-V4 | 16S_rv | TAAGAGACAGGACTACHVGGGTATCTAATCC |
| *H. sapiens* | actin | actin_fw | CACCATTGGCAATGAGCGGTTC |
| *H. sapiens* | actin | actin_rv | AGGTCTTTGCGGATGTCCACGT |

**Table S1.** *Quantitative PCR primers and targets for pathobiont-specific qPCR*
